# Characterization of quantitative susceptibility mapping in the left ventricular myocardium

**DOI:** 10.1016/j.jocmr.2024.101000

**Published:** 2024-01-17

**Authors:** Andrew Tyler, Li Huang, Karl Kunze, Radhouene Neji, Ronald Mooiweer, Charlotte Rogers, Pier Giorgio Masci, Sébastien Roujol

**Affiliations:** a School of Biomedical Engineering and Imaging Sciences, Kings College London, St Thomas’ Hospital, London, United Kingdom; b MR Research Collaborations, Siemens Healthcare Limited, Camberley, United Kingdom

**Keywords:** Cardiac, Myocardium, QSM, Iron, IMH, STEMI

## Abstract

**Background:**

Myocardial quantitative susceptibility mapping (QSM) may offer better specificity to iron than conventional T_2_* imaging in the assessment of cardiac diseases, including intra-myocardial hemorrhage. However, the precision and repeatability of cardiac QSM have not yet been characterized. The aim of this study is to characterize these key metrics in a healthy volunteer cohort and show the feasibility of the method in patients.

**Methods:**

Free breathing respiratory-navigated multi-echo 3D gradient echo images were acquired, from which QSM maps were reconstructed using the Morphology Enhanced Dipole Inversion toolbox. This technique was first evaluated in a susceptibility phantom containing tubes with known concentrations of gadolinium. *In vivo* characterization of myocardial QSM was then performed in a cohort of 10 healthy volunteers where each subject was scanned twice. Mean segment susceptibility, precision (standard deviation of voxel magnetic susceptibilities within one segment), and repeatability (absolute difference in segment mean susceptibility between repeats) of QSM were calculated for each American Heart Association (AHA) myocardial segment. Finally, the feasibility of the method was shown in 10 patients, including four with hemorrhagic infarcts.

**Results:**

The phantom experiment showed a strong linear relationship between measured and predicted susceptibility shifts (R^2^ > 0.99). For the healthy volunteer cohort, AHA segment analysis showed the mean segment susceptibility was 0.00 ± 0.02 ppm, the mean precision was 0.05 ± 0.04 ppm, and the mean repeatability was 0.02 ± 0.02 ppm. Cardiac QSM was successfully performed in all patients. Focal iron deposits were successfully visualized in the patients with hemorrhagic myocardial infarctions.

**Conclusion:**

The precision and repeatability of cardiac QSM were successfully characterized in phantom and *in vivo* experiments. The feasibility of the technique was also successfully demonstrated in patients. While challenges still remain, further clinical evaluation of the technique is now warranted.

**Trial Registration:**

This work does not report on a health care intervention.

## Graphical abstract

**Figure:**
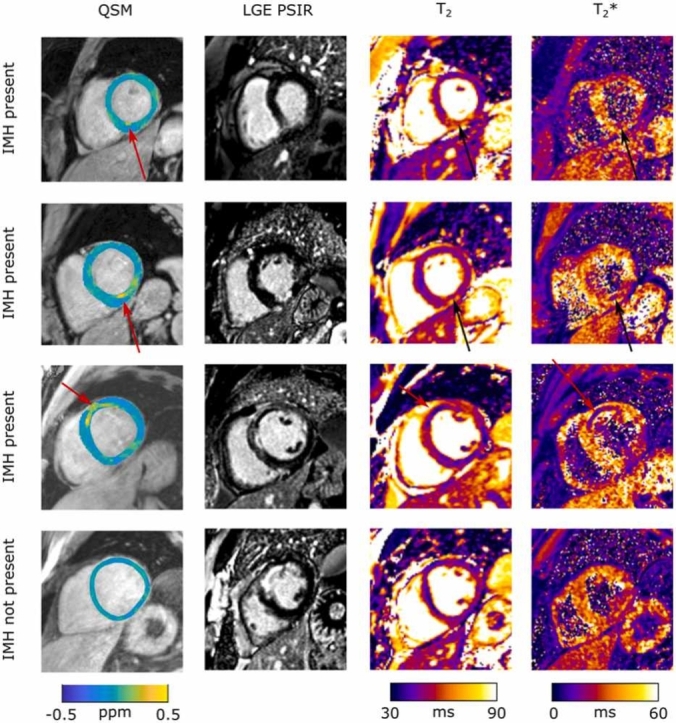

## Background

Myocardial quantitative susceptibility mapping (QSM) is a promising technique for the diagnosis and evaluation of several cardiac conditions involving excess iron [1]. One particular area of interest is the detection of the focal iron deposits found following a hemorrhagic myocardial infarction, which can give insight into the severity of the infarct and have a pathological role in adverse left ventricular remodeling [2], [3]. Current techniques for the detection of focal iron deposits in the myocardium include T_2_ [2], T_2_* [4], and T_1_ mapping [5]; however, each of these techniques has confounding factors [2], [5], [6], which may lead to missed diagnosis.

The principal potential benefit of QSM over T_1_, T_2_, and T_2_* mapping is its greater specificity to iron [6]. While T_1_/T_2_ is shortened by the presence of iron, factors associated with myocardial infarctions, such as intramyocardial fat (T_1_) or edema (T_1_/T_2_), among others can alter the relaxation times, confounding results [7], [8]. T_2_* is also influenced by a range of pathophysiological factors including the presence of edema, collagen, and fat which can confound the effect of iron, as demonstrated by Moon et al. [6]. Magnetic susceptibility, however, is influenced by these factors to a lesser extent, leading to maps which have the potential to better indicate iron concentration [6]. Despite this, the technique still requires detailed validation before it can be used in clinical research. In particular, its *in vivo* precision and repeatability have not yet been evaluated.

In this work, we present a series of experiments to characterize a myocardial QSM technique. Firstly, a phantom experiment was conducted to validate the accuracy of the technique against known susceptibility values. Secondly, a repeatability study, with 10 healthy volunteers, was performed to characterize the precision and repeatability of the technique. Finally, patient data, including five patients without expected focal iron deposition and five patients with a suspected intra-myocardial hemorrhage (IMH), were acquired in order to show the feasibility of the technique in patients.

## Quantitative susceptibility mapping theory

Magnetic susceptibility is a property which quantifies a material’s magnetic response to an external magnetic field. Magnetic susceptibility is commonly given relative to a standard material (in this case water) in units of parts per million (ppm), with a positive value indicating that the material is augmenting the magnetic field and a negative value that it is opposing the field. A material’s magnetic susceptibility depends on its molecular electronic configuration, with unpaired electrons generating a strong positive susceptibility and paired electrons a weak negative susceptibility. This means that metal ions, such as iron (II/III) or gadolinium (III), with several unpaired electrons, have strong positive shifts, allowing them to be visualized with QSM at concentrations seen in disease [1].

QSM aims to produce a map of susceptibility using magnetic resonance imaging (MRI). This is typically achieved by first measuring the magnetic field shift, using a multi-echo gradient echo (GRE) sequence, to produce a ∆B map, then inverting the equation,

(1) $$\Delta B\left(r\right)=B_{0}\left[d\left(r\right)⊛\chi \left(r\right)\right],d\left(r\right)=\frac{1}{4\pi }∙\frac{3\cos^{2}\theta -1}{{\left|r\right|}^{3}},$$

and finding the susceptibility *χ*(***r***). Unfortunately, $d\left(r\right)$, which is a dipole kernel, has two conical surfaces where $d\left(r\right)$ = 0, meaning that a simple inversion of the equation would result in a divide by zero, causing severe streaking artifacts. This is widely addressed by using iterative reconstruction methods with regularization [1], such as Morphology Enhanced Dipole Inversion (MEDI) [9] or total field inversion [10]. In this work, we used the MEDI reconstruction, due to its free availability and previous use in myocardial imaging [6]. The cost function, which is minimized by the MEDI reconstruction, is:

(2) $${\left\|\mathrm{MG}\chi \right\|}_{1}+\lambda \left({\left\|W\left(D\chi \left(r\right)-\Delta B\left(r\right)\right)\right\|}_{2}^{2}\right)$$

In the first term, *M* is a mask equal to one everywhere apart from edges determined from the magnitude images, and *Gχ*(*r*) is the gradient of the susceptibility map. This optimizes for smoothness in the calculated susceptibility map except on anatomical boundaries, where large changes are expected. In the second term, *λ* is the regularization parameter, *W* is proportional to the magnitude image, to account for noise variation, and *D* is the convolution form of the dipole kernel $d\left(r\right)$. This second term optimizes for the difference between the measured ∆*B*(*r*) map and the ∆*B*(*r*) map which would be predicted from *χ*(*r*).

While this approach allows for QSM to be performed without scans in multiple orientations [1], it does introduce an additional weighting parameter (λ).

## Methods

The accuracy of myocardial QSM was assessed with a purpose-built susceptibility phantom, containing tubes with a range of gadolinium concentrations. The *in vivo* precision and repeatability of the myocardial QSM pipeline were characterized in a cohort of 10 healthy volunteers. Feasibility of using the technique was then evaluated in a five-patient cohort. Five patients, with a ST-segment elevation myocardial infarction (STEMI) and suspected intramyocardial hemorrhage (IMH) were also scanned, to illustrate the potential the technique to detect focal regions of increased susceptibility (and therefore iron) *in vivo*.

### MRI scan protocol

All MRI acquisitions were performed using a 1.5T MRI scanner (MAGNETOM Aera and Sola (IMH patient 4), Siemens Healthineers, Erlangen, Germany) with human subjects in the supine position. All scans used a 32-element spine array coil and an 18-element body array coil (Siemens Healthineers, Erlangen, Germany).

The QSM acquisition consisted of a multi-echo 3D GRE research sequence. The acquisition parameters were five echoes, TE_1_ = 3.2 ms, ∆TE = 2.9 ms, field of view (FOV) = 288 × 384 × 100 mm^3^ (anterior-posterior × left-right × head-foot, phase encoding direction: anterior-posterior), voxel size = 1.5 × 1.5 × 5 mm^3^, flip angle = 15°, bandwidth = 543 Hz/Px, segments = 10, GRAPPA factor = 2, partial Fourier = 6/8. The shim box was set to tightly bound the heart and was carefully adjusted for each subject. Both magnitude and phase images were saved for each scan to allow QSM reconstruction.

### QSM reconstruction

**Fig. 1:**
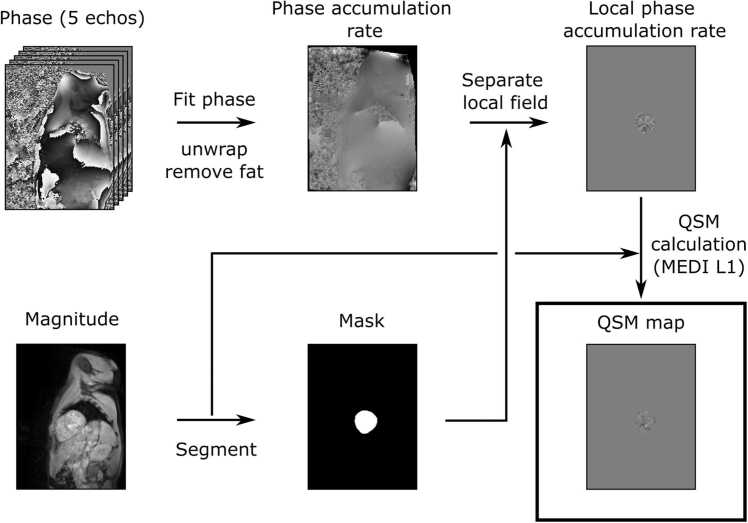
Illustration of the cardiac QSM reconstruction. Both magnitude and phase images are used in QSM reconstruction. A linear fit (against echo time) is applied to the phase images to calculate a map of rate of phase accumulation (total field). Phase wraps are removed from the total field map and fat signal removed with the SPURS algorithm. The magnitude images are segmented to produce a mask corresponding to the left ventricle, which allows the measured phase accumulation rate to be separated into two components, one resulting from local susceptibility shifts, originating from within the left ventricle and the other from background susceptibility shifts outside of the left ventricle. The QSM map is finally calculated from this local phase accumulation rate map using the MEDI L1 algorithm, which incorporates further prior morphological information into the reconstruction from the magnitude images. QSM: quantitative susceptibility mapping, MEDI: Morphology Enhanced Dipole Inversion.

QSM maps were reconstructed using the MEDI toolbox (version January 15, 2020) [9], [11], [12], [13], [14] in MATLAB (version R2019b, MathWorks, Natick, Massachusetts) ( Fig. 1). Cardiac maps were reconstructed with a region of interest (ROI) corresponding to the left ventricle only, to reduce susceptibility artifacts in the septum (Supplementary Figs. 1 and 2). The total field was found with a linear fit to the phase images and processed with the simultaneous phase unwrapping and removal of chemical shift (SPURS) algorithm (including T_2_*- iterative decomposition of water and fat with echo asymmetry and least squares estimation [IDEAL]) to simultaneously unwrap the total field and remove the fat signal [15]. The local field shift was then extracted with the projection onto dipole fields algorithm [11], [16]. For the MEDI reconstruction, the optimal regularization parameter was assessed as λ = 100, as described in the phantom and healthy volunteer experiment sections, which was then used for the remaining analysis. The local field shift and mask arrays input into MEDI were zero padded in the z-dimension to give an FOV of 288 × 384 × 335 mm^3^_._ Spherical mean value smoothing was not used.

### Phantom experiments

The phantom experiments utilized a gadolinium phantom consisting of six 50 mL centrifuge tubes (Corning, Corning City, New York) in a water bath. Each tube contained a solution with different concentrations of gadobutrol (Bayer, Leverkusen, Germany). The concentrations were chosen to span the range of physiological magnetic susceptibilities arising from excess iron [6] and include higher values to assess the effect of streaking artifacts at higher susceptibility shifts. The concentrations were 0.0, 0.6, 1.5, 3.1, 6.1, 9.2 mM/dm^3^ giving theoretical susceptibilities of 0.0, 0.2, 0.5, 1.0, 2.0, 3.0 ppm, respectively, when calculated with the Curie law [17]. The shim-box and reconstruction ROI were set to the volume of the water bath. The mean and standard deviation of the voxels within an ROI defined for each tube in a central slice (to avoid the large susceptibility shift relative to water, of the acrylic tube stand which held the tubes at the top and bottom) of the resultant susceptibility map were calculated, and fit with a linear model of measured against predicted susceptibility. λ values of 50, 100, 250, and 500 were used for the experiment and L-curve analysis was performed using the zero curvature approach [18] across a range of λ values from 0.1 to 1 × 10^7^.

### *In vivo* characterization in healthy volunteers

*In vivo* precision and repeatability were characterized with a test-retest procedure. The cohort consisted of 10 healthy volunteers (5M/5F, body mass index (BMI) = 25 ± 4 kg/m^2^, age = 31 ± 7 years). Each subject was scanned for two sessions, with a short break outside of the scan room between sessions. Each session consisted of anatomical localizers (acquired independently from the other session), followed by the free breathing multi-echo 3D GRE acquisition (mean duration = 5.5 ± 2.0 minutes) required for QSM in short axis orientation. The localizers were acquired during breath holds (exhalation). The multi-echo GRE acquisition was free-breathing and respiratory-navigated with a diaphragmatic navigator [19] (gated to exhalation, window = 4 mm, mean efficiency = 51 ± 12%, no slice tracking) and electrocardiogram triggered (mid-diastolic rest period).

The QSM results were analyzed using the American Heart Association (AHA) 16 segment model [20], with the reconstructed cardiac slices sorted into apical, mid, and basal groups, which were then used for the three slices of the AHA model. Mean segment susceptibility, precision (standard deviation of voxel magnetic susceptibilities), and repeatability (absolute difference in segment mean susceptibility between repeat 1 and 2) were calculated for each myocardial segment. This procedure was repeated for reconstructions of the dataset with λ values of 50, 100, 250, and 500, and the mean segment susceptibility, precision, and repeatability, for each lambda value, were plotted to quantify the effect of the regularization parameter.

### *In vivo* characterization in patients

Ten patients, referred for clinical cardiovascular magnetic resonance (CMR) late gadolinium enhancement (LGE), were scanned with the multi-echo GRE sequence (mean navigator efficiency = 48 ± 14%,mean duration = 5.8 ± 2.0 minutes), described in the healthy volunteer cohort section, in addition to their regular CMR exam including LGE phase sensitive inversion recovery (PSIR) images and T_2_ mapping imaging. Of these, five (2M/3F, BMI = 29 ± 3, age = 48 ± 20 years) were not expected to exhibit focal iron deposits (patients indicated with: atrial fibrillation, amyloidosis, hypertension, left ventricular (LV) systolic dysfunction, and dilated cardiomyopathy), and five had an STEMI with suspected IMH (4M/1F, BMI = 30 ± 7, age = 50 ± 11 years). The susceptibility maps for the group of five non-infarct patients were reconstructed with λ = 100, and the resultant maps were analyzed with the AHA 16 segment model [20]. For this group, both segment means and precision were calculated. Susceptibility maps for one STEMI patient with IMH were reconstructed with λ values of 50, 100, and 250. Additionally the resulting QSM maps of this patient were compared to LGE images and T_2_ maps (matched T_2_* map not available). Susceptibility maps for the four remaining STEMI patients were reconstructed with λ = 100, and the maps compared to LGE images, T_2_, and T_2_* maps.

## Results

### Phantom experiments

**Fig. 2:**
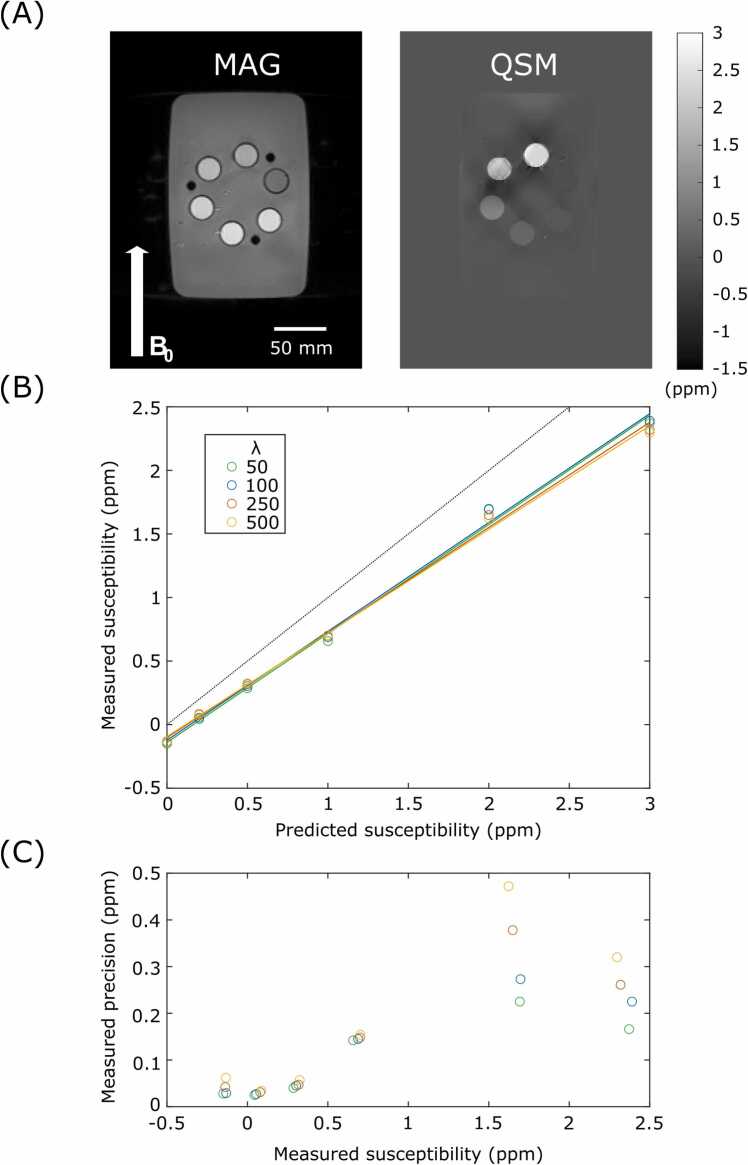
Phantom experiments. (A) Magnitude image (MAG) and susceptibility map (QSM) of the magnetic susceptibility phantom. (B) Plot of the measured magnetic susceptibility, against predicted susceptibility. All the linear fits had R^2^ > 0.99. (C) Plot of measured precision against measured susceptibility for all tubes and λ values. QSM: quantitative susceptibility mapping.

The results of the phantom experiments are shown in Fig. 2. λ in the range of 100 to 250 was found to be optimal by L-curve analysis, with minor streaking artifacts only visible in the two highest susceptibility tubes, both of which are above the physiological range, for λ = 100. All values of λ gave a strong linear relationship with the expected shift calculated from the concentration of gadolinium contrast agent (R^2^ > 0.99). For λ = 100, the relationship was of the form $\chi_{m}$ = 0.85$\chi_{p}$ − 0.12, where $\chi_{m}$ and $\chi_{p}$ are the measured and expected susceptibility shifts, respectively. Although measured susceptibility was not largely affected by λ, there was a greater effect on precision, with reduced regularization (greater λ) leading to relatively poorer precision, particularly in tubes with greater gadolinium concentrations.

### *In vivo* characterization in healthy volunteers

**Fig. 3:**
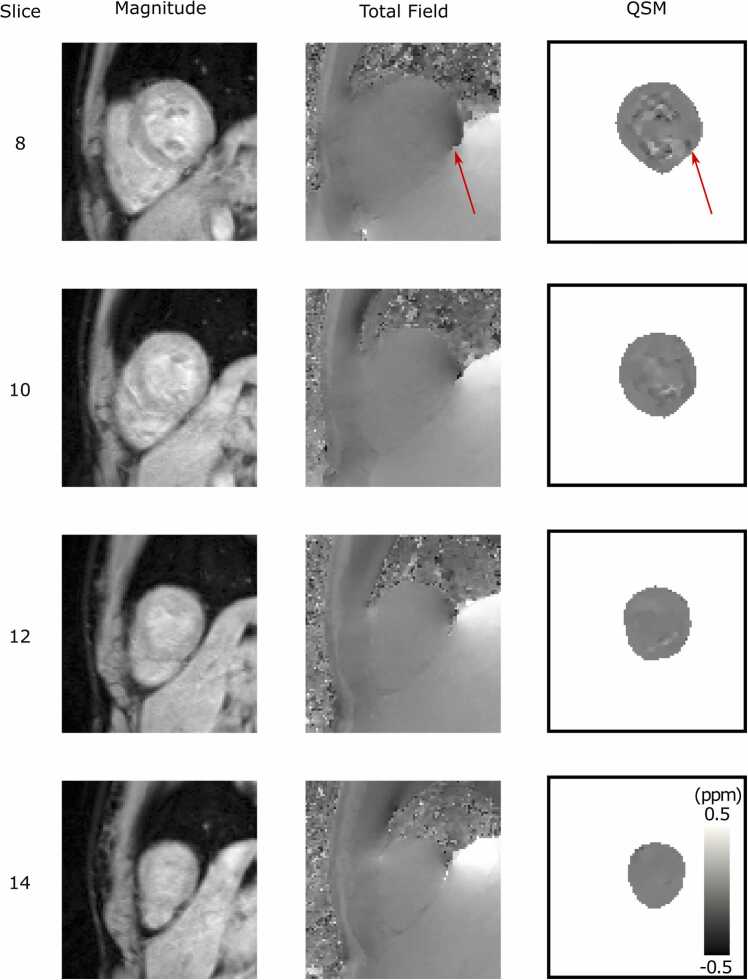
*In vivo* cardiac QSM in one healthy volunteer. Example magnitude and fitted total field short axis images with their corresponding QSM map (λ = 100). Artifactual signal, due to a shim defect at the heart-lung-liver interface is highlighted with a red arrow. Note that magnitude and field images do not share the same scale with QSM maps. ppm: parts per million, QSM: quantitative susceptibility mapping.

Example susceptibility maps for one volunteer are shown in Fig. 3 alongside the acquired magnitude and phase images. The importance of shim quality (limited by the homogeneity of the B_0_ field and available shim sets) is clearly demonstrated, with the region at the heart-lung-liver interface (red arrow), where the shim quality is lower (see phase image), showing an artifactual region of lower susceptibility. Inhomogeneity within the blood pool is also visible, particularly in slices 8 and 10, illustrating the effect of the edge mask (M) which is influenced by the strong intensity gradient in the magnitude images caused by the papillary muscles.

**Fig. 4:**
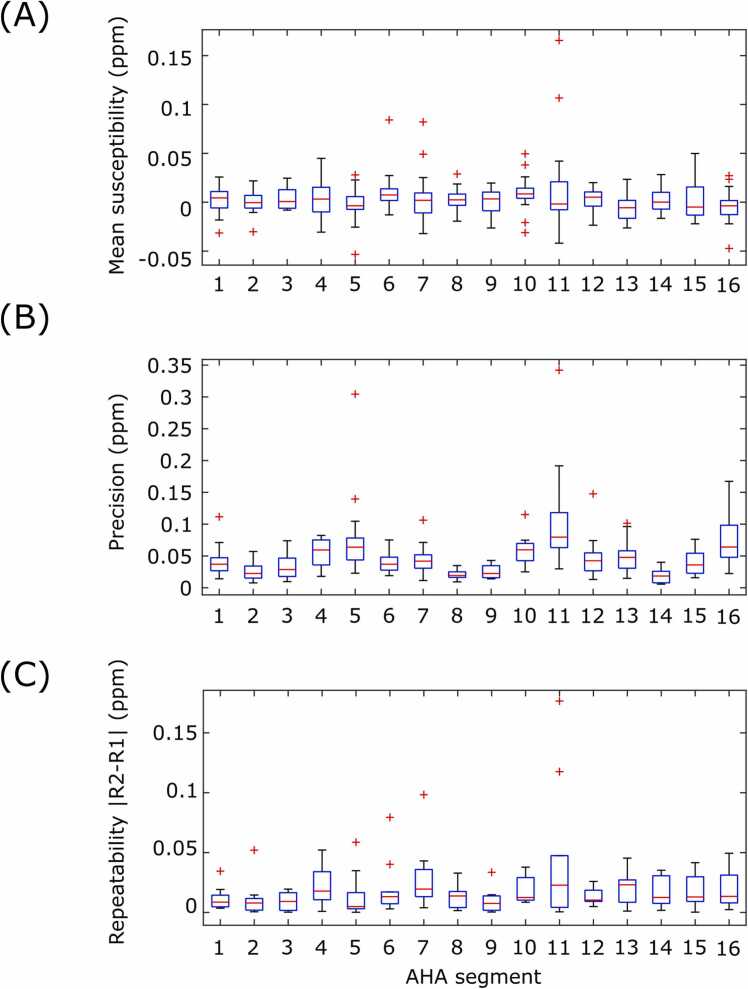
Characterization of cardiac QSM in healthy volunteers (λ = 100). (A) AHA segment mean susceptibility. (B) AHA segment precision (spatial standard deviation). (C) Repeatability (absolute difference in susceptibility between repeats) for each AHA segment. AHA: American Heart Association, ppm: parts per million, QSM: quantitative susceptibility mapping.

**Fig. 5:**
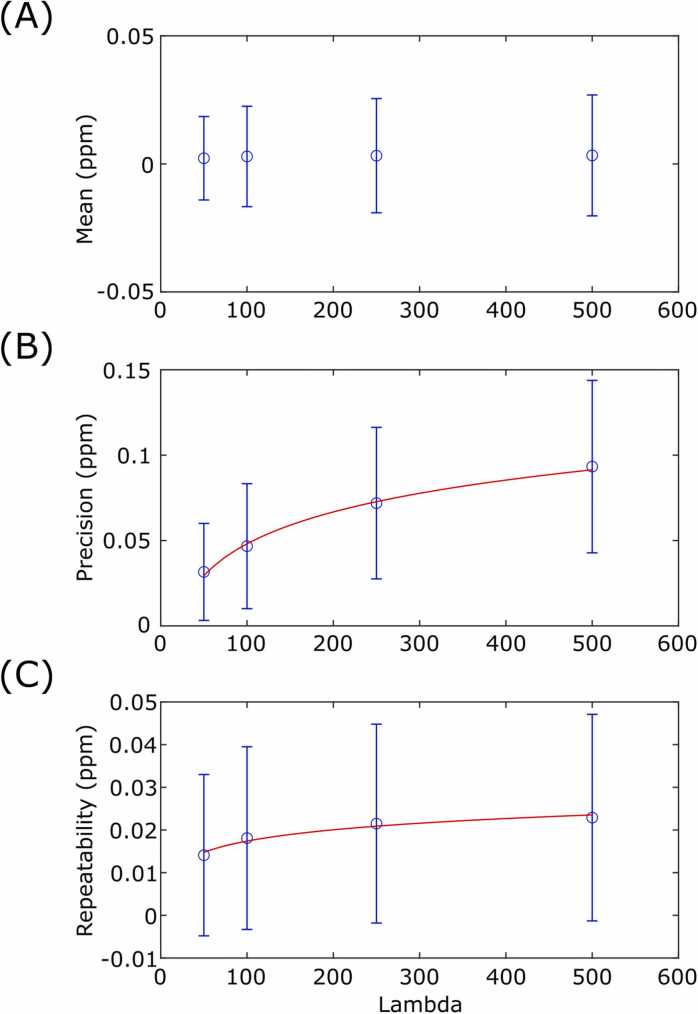
Influence of the regularization parameter for *in vivo* cardiac QSM in healthy volunteers. Mean (A), precision (B), and repeatability (C) of the magnetic susceptibility in the healthy volunteer cohort, for reconstructions with a range of regularization levels (in this case, higher λ reduces the degree of regularization). ppm: parts per million, QSM: quantitative susceptibility mapping.

Quantitative results are shown in Figs. 4 and 5. With λ = 100, the mean segment susceptibility was 0.00 ± 0.02 ppm, and the precision was 0.05 ± 0.04 ppm. The mean absolute difference in segment means between repeats at λ = 100 was 0.02 ± 0.02 ppm. The effects of susceptibility artifacts at the heart-lung-liver interface were present in the quantitative results, with AHA segments 4/5 and 10/11 displaying the highest (worst) precision values for the basal and mid myocardium, respectively. The segment mean susceptibility was minimally effected by λ (*χ*(λ) = 0.00046 log(λ) + 0.00059, R^2^ = 0.87); however, both the precision and repeatability showed linear-log relationships, where decreasing λ (greater regularization) led to improved (numerically lower) precision and repeatability. The fit of the precision (P) was P(λ) = 0.0269 log(λ) – 0.076 (R^2^ = 0.996), and the fit of repeatability (R) was R(λ) = 0.0038 log(λ) − 0.0001 (R^2^ = 0.963).

### *In vivo* characterization in patients

**Fig. 6:**
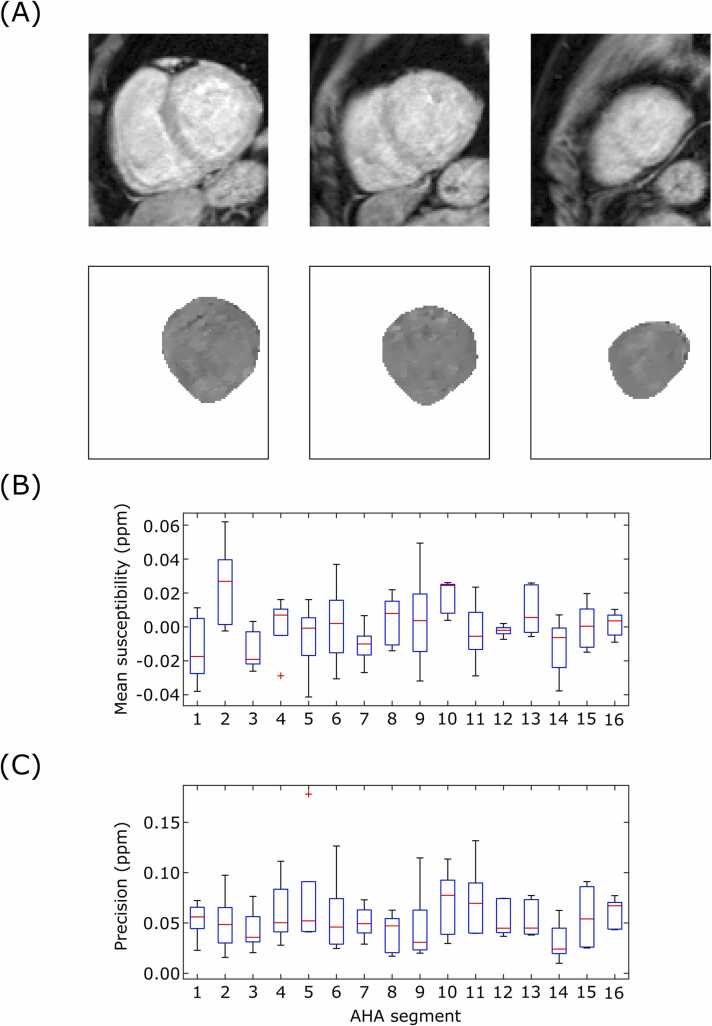
Characterization of cardiac QSM in patients without IMH (λ = 100). (A) Example images of one patient showing basal, mid, and apical slices of a cardiac QSM map. (B) AHA segment mean susceptibility. (C) Precision (spatial standard deviation) of AHA segment. AHA: American Heart Association, IMH: intramyocardial hemorrhage, ppm: parts per million, QSM: quantitative susceptibility mapping.

The results of the five non-infarct patient cohort are shown in Fig. 6. The susceptibility of the myocardium appears visually similar to the healthy volunteer cohort. With λ = 100, the mean segment value was 0.00 ± 0.02 ppm and precision was 0.05 ± 0.03 ppm. There was no significant difference to the healthy volunteer cohort (Welch’s t-test, p = 0.79 and 0.62, for segmental means and precision, respectively).

**Fig. 7:**
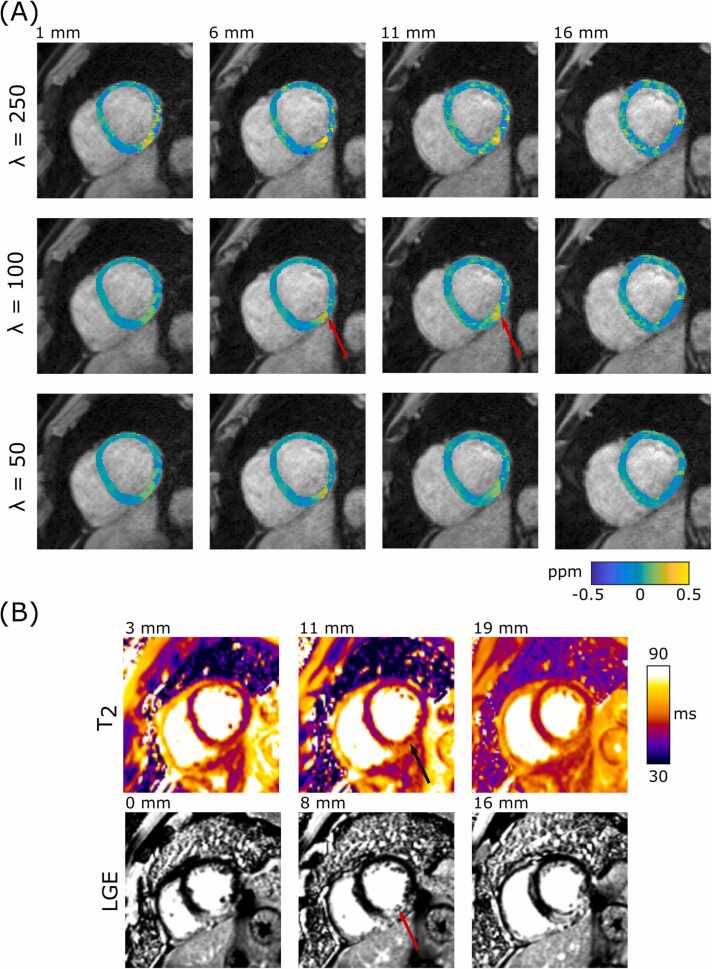
(A) Susceptibility maps for a patient exhibiting an IMH (patient 1). There is a clear focal susceptibility elevation in the myocardium, (red arrow) which is consistent with the IMH seen in the LGE images and T_2_ maps. Increased regularization (lower λ) reduced noise in the map, however the peak signal intensity of the hemorrhage was also reduced. (B) T_2_ and LGE PSIR images of the infarct. T_2_ maps show a large hyper-intense region of edema in the infero-lateral position, with a small hypo-intense area of IMH (black arrow). The LGE PSIR images show a transmural hyper-intense scar in the infero-lateral position with a hypo-intensity (red arrow), indicating disruption to the microvasculature, co-located with the hemorrhage seen on the T_2_ map. Relative slice proscribed position is indicated above each image. IMH: intramyocardial hemorrhage, LGE: late gadolinium enhanced, PSIR: phase sensitive inversion recovery.

Fig. 7 shows reconstructed QSM, LGE, and T_2_ mapping images from a patient with a significant, transmural, STEMI. The T_2_ maps show a large hyper-intense region of edema in the infero-lateral position, with a small hypo-intense area of IMH. The LGE images show a transmural hyper-intense scar in the infero-lateral position with a hypo-intensity, indicating disruption to the microvasculature, co-located with the IMH seen on the T_2_ map. At λ = 100, a hyper-intense region is clearly visualized on the QSM map, with regions outside of the hyper-intense region not showing significant susceptibility artifacts. The hyper-intense region coincides with the IMH seen on the T_2_ map, suggesting sensitivity to iron deposition from hemorrhagic infarcts. Higher λ values appear to have greater artifact content and noise, as expected from the healthy volunteer cohort, while the peak intensity of the IMH reduces with lower λ (increased regularization).

**Fig. 8:**
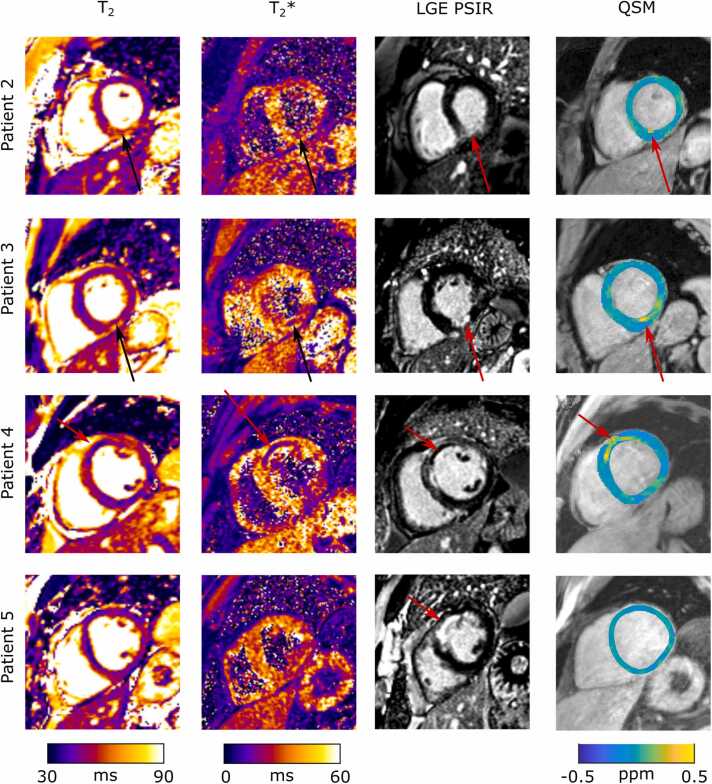
**:** LGE, T_2_, T_2_*, and QSM maps for four patients with suspected IMH. All patients show a significant infarct on LGE images. In patients 2–4, the T_2_ maps show a hyper intense region of edema, containing hypo intense areas, indicative of IMH. In these patients the T_2_* maps show hypo intense regions, on the endocardial wall, indicating iron deposition associated with IMH. In patient 5, the T_2_ and T_2_* maps do not indicate iron deposition. QSM maps are in agreement with T_2_/T_2_* maps with hyper intense regions visible within the infarct in patients 2–4. IMH: intra-myocardial hemorrhage, LGE: late gadolinium enhanced, ppm: parts per million, QSM: quantitative susceptibility mapping.

Fig. 8 shows LGE, T_2_, T_2_*, and QSM maps for four further patients presenting with STEMI and suspected IMH. The scars are visualized in the LGE images, with the associated edema visualized in the T_2_ map. The T_2_/T_2_* maps indicate IMH in three of the cases with hypo intense regions visible within the scar. The QSM maps with λ = 100 show clear hyper intense regions, co-located with hypo intense regions on the T_2_* maps for the three cases where IMH is detected with the T_2_/T_2_* maps. In the final case (patient 5), no IMH was identified with QSM or T_2_/T_2_* mapping, and no evidence of microvascular obstruction was seen on the LGE images.

## Discussion

The accuracy, precision, and repeatability of cardiac QSM in the left ventricle were characterized using phantom and *in vivo* experiments with a range of regularization parameters. Accuracy was confirmed with phantom experiments, which showed a strong correlation between measured magnetic susceptibilities and contrast agent concentrations in the phantom. Precision and repeatability were quantified in a cohort of 10 healthy volunteers, and feasibility was confirmed in a patient cohort, including five patients with suspected IMH.

The results of the phantom experiments demonstrated that it is possible to accurately quantify magnetic susceptibility with this QSM method. All λ values returned an R^2^ of greater than 0.99, for the linear fit of measured susceptibility against predicted susceptibility, indicating an excellent correlation between the measured, and actual susceptibility of the solution in the tubes. The results also showed that although the regularization parameter used in the reconstruction had little impact on the measured susceptibility, at least for homogeneous objects with a clear edge; however, there was a measurable impact on precision. This is as expected from literature [21], and likely due to the incorporation of the edge mask into the reconstruction, which prevents penalization of sharp susceptibility variation between regions separated by an edge in the optimization, while still penalizing variation within regions. There was a strong linear relationship between the predicted magnetic susceptibility shift (based on the Curie law) and measured shift, although the line of best fit did show a non-zero offset and gradient of less than one. This has been observed previously [9] however, and in this case the zero-offset may be due to the close proximity of the tubes, resulting in the streaking artifact from one tube overlapping with its neighbors, or potentially imperfect background field removal.

The results of the healthy volunteer cohort study show both the promise of QSM in the myocardium and the challenges, which still need to be overcome. The segment mean across all scans was close to zero (0.00 ± 0.02 ppm), as expected for a healthy volunteer cohort, and the precision was 0.05 ± 0.04 ppm. In the work by Moon et al. [6], a cohort of seven STEMI patients were scanned, of those four had IMH with a susceptibility of 0.16 ± 0.09 ppm within the infarct, compared to −0.01 ± 0.06 ppm in the remote region of all seven patients. This suggests that the precision of the procedure would have been sufficient to measure the iron deposition in those cases; however, smaller myocardial hemorrhages may not be visible above the noise/artifact level, potentially decreasing the utility of the technique for risk stratification in more borderline cases. Importantly, from the perspective of developing a useful clinical tool, the measured repeatability (0.02 ± 0.02 ppm) was in line with the precision (0.05 ± 0.04 ppm), indicating that the procedure is not very sensitive to exact patient position/scan planning or inter-scan physiological variation, such as heart/respiratory rate. Despite these promising results, and the generally smooth *in vivo* myocardial susceptibility maps, there was variation in the precision and repeatability between the AHA segments. This is likely caused by a susceptibility artifact at the heart-lung-liver interface resulting from poor shim.

When the healthy volunteer dataset was reconstructed with a range of values for the regularization parameter, this had minimal effect on the AHA segment mean values; however, we did observe a linear-log relationship to both precision and repeatability, reflecting the smoothing effect of the regularization term. Although the effect of regularization on the segment means was minimal, this result does not show that changing the regularization of the reconstruction will not affect the diagnostic accuracy of the technique in diseased patients. In particular, focal hyper-intensities could be smoothed out by greater regularization and missed, or too little regularization could lead to false positives from artifacts, making selection of the correct value of regularization parameter critical. Further clinical studies in disease patients are required to confirm if the currently optimized regularization parameter remains optimal.

Following on from the healthy volunteer experiments, we confirmed the feasibility of the employed myocardial QSM procedure in a cohort of 10 patients, of these five were not expected to have focal iron deposits and five had suspected IMH. The susceptibility and precision of the five patients were not significantly different from the healthy control cohort, indicating that the technique was not unduly adversely affected by the differences between patient and healthy volunteer cohorts, and providing confidence in the applicability of the healthy volunteer cohort’s results to patients.

The suspected IMH cases presented in this work illustrate the feasibility of this technique to resolve the focal regions of elevated magnetic susceptibility associated with iron deposits. The first IMH case also illustrates the impact of λ on the identification of focal susceptibility hotspots. In this case, reducing λ (increased regularization) reduced the level of noise in the susceptibility maps (as expected from the healthy volunteer cohort); however, it also reduced the peak susceptibility of the IMH itself, potentially suggesting lower sensitivity. This further illustrates the potentially important limitation of the MEDI reconstruction for this application, where the infarct was not visualized in the GRE magnitude images, and therefore the susceptibility originating from the focal iron signal was dampened by regularization. One approach to resolving this potential limitation may be to use an AI-based reconstruction method [22] which may be able to extract more information from the multi-echo GRE dataset than the edge mask prior information used by MEDI. In the final four cases, both T_2_* and QSM maps were acquired. In each case, there was good agreement between the iron deposits identified by each method, including in one case where no iron/IMH was detected. In all cases, some degree of edema was observed in the scar region, which could potentially obscure a small IMH. However, the negative case was completely negative for IMH on the T_2_ map and T_2_* imaging. Moreover, no microvascular obstruction was seen on the LGE images. Overall, this illustrates the potential utility of QSM for identifying patients who could benefit from novel therapies for removal of pathological iron from the myocardium [3].

## Limitations

The study has several limitations. First, all data were acquired at 1.5T. Therefore, further study will be necessary to characterize this technique at different field strengths. Second, all repeatability data were acquired on the same scanner and repeatability of QSM across different scanner types from different vendors remains to be investigated. Finally, the patient study had a small sample size and was only used to study feasibility. A large-scale study enrolling patients with a range of cardiac diseases, including those with expected iron deposition (IMH and thalassemia) and those without is now warranted.

## Conclusion

Left ventricular myocardial QSM was successfully characterized in phantom and *in vivo* experiments. The *in vivo* repeatability and precision of QSM in the left ventricular myocardium suggest this method has potential for different clinical scenarios, including iron-level assessment in patients with an STEMI. Further clinical evaluation of the technique is now warranted.

## Funding

Biomedical Research Centre at Guy's and St Thomas' National Health Service (NHS) Foundation Trust; 10.13039/501100000274British Heart Foundation (BHF), Grant/Award Numbers: (PG/19/11/34243), (PG/21/10539); 10.13039/501100000266Engineering and Physical Sciences Research Council (EPSRC), Grant/Award Number: (EP/R010935/1); King's College London; Innovate UK, Grant/Award Number: (68539); 10.13039/501100000272National Institute for Health Research (NIHR). This research was funded in whole, or in part, by the 10.13039/100004440Wellcome Trust (WT 203148/Z/16/Z). For the purpose of open access, the author has applied a CC BY public copyright licence to any Author Accepted Manuscript version arising from this submission.

## Authorship contribution

**Tyler Andrew:** Writing – review and editing, Writing – original draft, Software, Methodology, Investigation, Formal analysis. **Roujol Sebastien:** Writing – review and editing, Writing – original draft, Supervision, Methodology, Investigation, Funding acquisition, Conceptualization. **Masci Pier Giorgio:** Writing – review and editing, Methodology, Conceptualization. **Rogers Charlotte:** Writing – review and editing, Investigation. **Mooiweer Ronald:** Writing – review and editing, Investigation. **Neji Radhouene:** Writing – review and editing, Software. **Kunze Karl:** Writing – review and editing, Software. **Huang Li:** Writing – review and editing, Software.

## Ethics approval and consent

All healthy volunteers and patient participants gave informed written consent for this study, which had separate research ethics committee approval for both healthy volunteer and patient cohorts (approval number 01/11/12 for the healthy volunteer study and approval number 15/NS/0030 for the patient study), and was in full compliance with the Declaration of Helsinki.

## Consent for publication

Consent for publication was obtained from all participants in the study.

## Declaration of competing interest

The authors declare the following financial interests/personal relationships which may be considered as potential competing interests: L.H. reports financial support was provided by Neoscan Solutions GmbH. P.G.M. reports a relationship with Perspectum Ltd. that includes consulting or advisory. L.H.: KCL employee at the time of the study and has since become an employee of Neoscan Solutions GmbH. K.K. and R.N.: Employees of Siemens Healthcare Limited. R.M.: Seconded to Siemens Healthcare Limited. P.G.M.: Consultant for Perspectum Diagnostics Limited. If there are other authors, they declare that they have no known competing financial interests or personal relationships that could have appeared to influence the work reported in this paper.
