# Supplementary material for: Characterization of quantitative susceptibility mapping in the left ventricular myocardium
Source: J Cardiovasc Magn Reson. 2024 Jan 17;26(1):101000. doi: 10.1016/j.jocmr.2024.101000 (PMC11129096; doi:10.1016/j.jocmr.2024.101000)

**Supplementary Information**


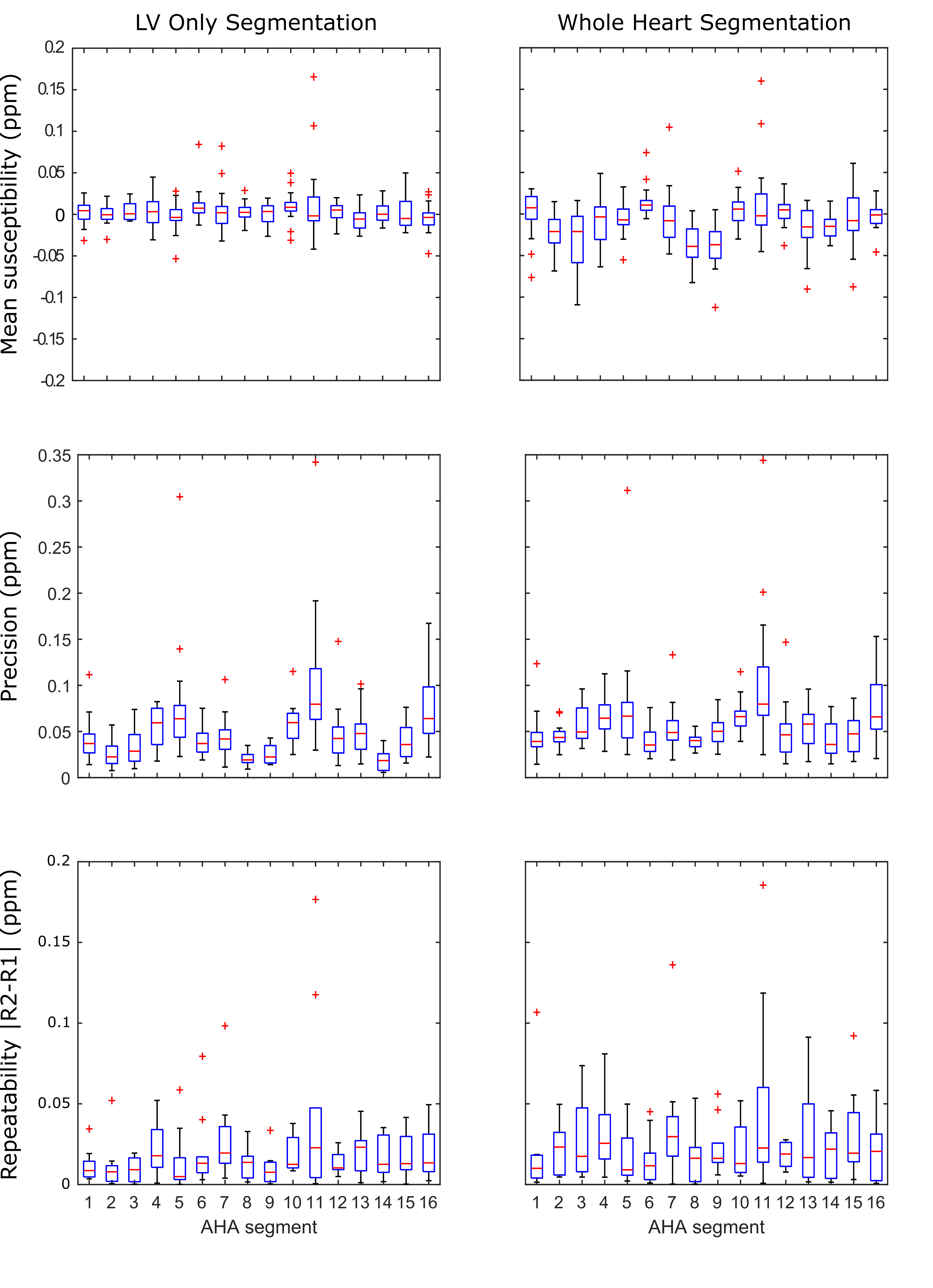


**Supplementary Information Figure 1:** Boxplots of mean, precision and repeatability for LV only and whole heart segmentation of the healthy volunteer cohort. LV only: mean = 0.00 ± 0.02 ppm, precision = 0.05 ± 0.04 ppm, repeatability = 0.02 ± 0.02 ppm. Whole heart: mean = -0.01 ± 0.03 ppm, precision = 0.06 ± 0.03 ppm, repeatability = 0.03 ± 0.03 ppm.

**Supplementary Information Figure 2:** Reconstructed QSM maps for IMH patient 2, with whole heart and LV only masks. A negative susceptibility artefact can be seen in the septum (red arrows). The haemorrhage can be seen in both reconstructions (black arrow).
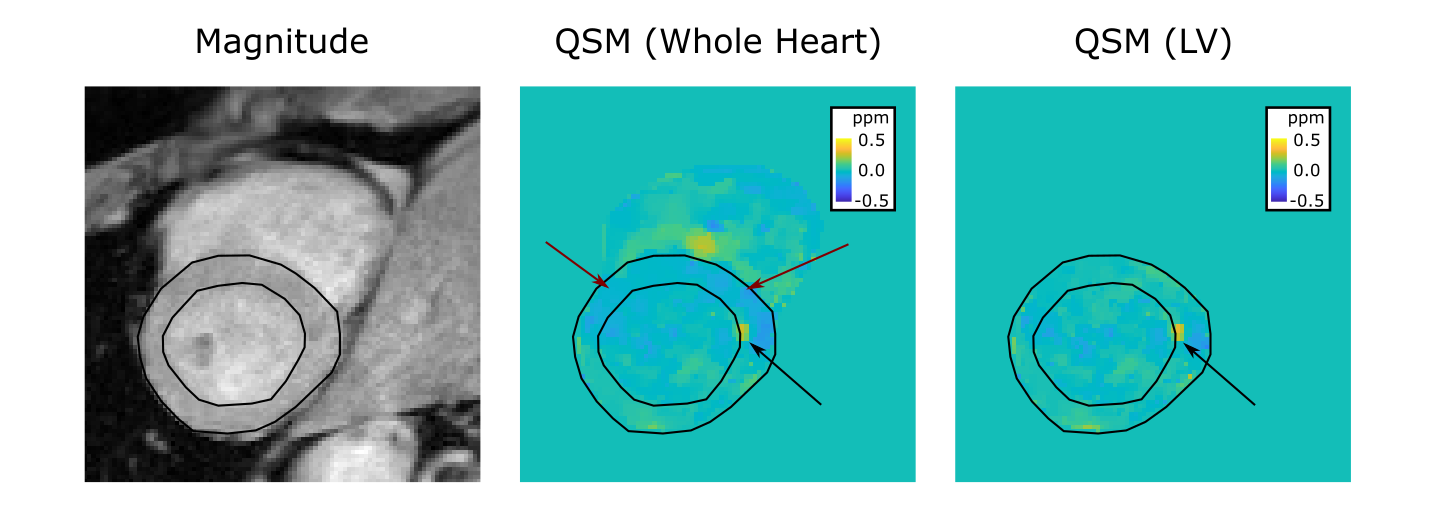

Supplement: Supplementary file 1 — Supplementary material [file mmc1.docx]
